# Estetrol and Mammary Gland: Friends or Foes?

**DOI:** 10.1007/s10911-021-09497-0

**Published:** 2021-08-31

**Authors:** Anne Gallez, Isabelle Dias Da Silva, Vincent Wuidar, Jean-Michel Foidart, Christel Péqueux

**Affiliations:** Laboratory of Biology, Tumors and Development, GIGA-Cancer, University of Liège, Liège, Belgium

**Keywords:** Estetrol, Estrogen receptor, Mammary gland, Breast cancer, Menopause, Contraception

## Abstract

Estrogens have pleiotropic effects on many reproductive and non-reproductive tissues and organs including the mammary gland, uterus, ovaries, vagina, and endothelium. Estrogen receptor α functions as the principal mediator of estrogenic action in most of these tissues. Estetrol (E4) is a native fetal estrogen with selective tissue actions that is currently approved for use as the estrogen component in a combined oral contraceptive and is being developed as a menopause hormone therapy (MHT, also known as hormone replacement therapy). However, exogenous hormonal treatments, in particular MHTs, have been shown to promote the growth of preexisting breast cancers and are associated with a variable risk of breast cancer depending on the treatment modality. Therefore, evaluating the safety of E4-based formulations on the breast forms a crucial part of the clinical development process. This review highlights preclinical and clinical studies that have assessed the effects of E4 and E4-progestogen combinations on the mammary gland and breast cancer, focusing in particular on the estrogenic and anti-estrogenic properties of E4. We discuss the potential advantages of E4 over current available estrogen-formulations as a contraceptive and for the treatment of symptoms due to menopause. We also consider the potential of E4 for the treatment of endocrine-resistant breast cancer.

## Introduction

Estrogens are master drivers of reproductive activity in women; controlling ovulation, zygote implantation, and mammary gland development [1–3]. Estrogens also have pleiotropic protective effects on non-reproductive tissues and organs and help to ensure optimal health of women during their childbearing years [4–10]. A women’s life can be divided into five main stages based on estrogen activity: childhood, puberty, reproductive life, perimenopause and postmenopause. Estrogen levels increase during puberty until menarche, when levels stabilize. They then vary in line with the monthly menstrual cycle until late perimenopause, when estrogen levels fall and stabilize. Menopause hormone treatments (MHTs), also known as hormone replacement therapy (HRT), are the most powerful treatments available for alleviating the symptoms associated with the cessation of estrogen production by the ovaries at menopause [11, 12]. However, these estrogen-based treatments are associated with an increased risk of breast cancer and estrogens also act as growth factors in estrogen receptor-positive (ER +) breast cancers, which account for 70% of all cases [13]. Therefore, there is an unmet medical need for a new generation of MHT with an improved benefit/risk profile for breast cancer in particular.

The ideal estrogen would display the following characteristics: 1) effective on menopause symptoms (in particular hot flushes, vulvo-vaginal atrophy, and osteoporosis); 2) neutral on mammary gland and breast cancer growth; 3) neutral on endometrial hyperplasia, which increases the risk of endometrial cancer; 4) cardioprotective against atherosclerosis and thromboembolism; 5) a favorable metabolic profile.

Accumulating data support the use of estetrol (E4), a natural fetal estrogen, for such clinical applications. E4 is currently approved by the US Food and Drugs Administration and by the European Medicines Agency as the estrogen component in a combined oral contraceptive and is under development for the treatment of symptoms due to menopause. It is also being evaluated for advanced endocrine-resistant breast cancer. In this review, we aim to define the extent to which E4 fulfills ‘the ideal criteria’, focusing in particular on its effects on the mammary gland and breast cancer. We will provide a brief summary of the clinical evidence associated with breast cancer risk and the uptake of exogenous estrogen-based formulations, followed by a review of E4 biology, E4 signaling pathways, and its actions on the mammary gland and breast cancer. We will then move on to discuss the potential benefits of E4 as a contraceptive, as a treatment for symptoms due to menopause, and for the treatment of endocrine-resistant breast cancer.

## Estrogen-based Formulations and Breast Cancer Risk

Estrogens are widely used as a hormonal treatment for multiple therapeutic indications, in particular for contraception and in the treatment of symptoms due to menopause. Currently, 17β-estradiol (E2) and estriol (E3) are the most widely prescribed natural estrogens in combined oral contraceptives (COCs) and in MHT [11, 12]. Synthetic estrogenic molecules such as conjugated equine estrogen, ethinyl estradiol and E2-valerate are also used [12, 14].

Over the past two decades, the safety of COCs on breast tissue has been debated in the literature and overall, the risk of COC-associated breast cancer risk appears to remain low. However, there is limited evidence available as the incidence is low, studies are difficult, and require very large cohorts of patients [15–17]. Interestingly, the slight increase in risk that is observed, disappears ten years or more after treatment cessation [15], highlighting that estrogens promote preexisting breast cancer cell growth rather than inducing breast carcinogenesis. Moreover, the risk of ovarian, endometrial, and colorectal cancers is reduced for women using COCs [18], and such advantages may outweigh the potential negative effect of COCs on breast cancer risk for premenopausal women.

In 2002, for the first time, the Women’s Health Initiative (WHI) study [19] reported an association between MHT use and breast cancer risk. This study had unprecedented consequences on the prescription rates of MHTs, which subsequently decreased by 30% [20]. Between 2003 and 2011, several European and American epidemiological and observational studies including, the Million Women Study, the French E3N cohort, a study of Finnish women, and the European Prospective Investigation into Cancer and Nutrition (EPIC) studies, referenced breast cancer risk as a major side effect of MHTs [19, 21–25]. In 2012, the Cochrane meta-analysis confirmed the pro-tumoral risk of MHT [26] and in 2019, a long-term prospective study, with a mean follow-up period of 17.6 years, showed a correlation between MHT and breast cancer-associated mortality [27]. Finally, a meta-analysis of 58 studies, including 143,887 postmenopausal women with breast cancer and 424,972 women without breast cancer, was published by the Collaborative Group on Hormonal Factors in Breast Cancer [28] and again confirmed the correlation between MHT use and breast cancer risk. However, variations in the relative risk ratio were reported depending on modality (Table 1) and all MHT formulations, with the exception of estrogenic vaginal cream, were associated with an increased risk [28]. An excess risk of breast cancer was associated with both current or recent use (1–4 years) and long-term treatment. The incidence of mammary cancers correlated with treatment duration [23, 29] and decreases progressively after treatment cessation [21, 29]. The association between breast cancer and MHT use was higher for estrogen-progestogen combinations compared to estrogen-only formulations or placebo [12, 21, 24–26, 28]. Combinations with natural progesterone and dydrogesterone appeared to be safer [22] than those combined with synthetic progestins such as norethisterone acetate and medroxy-progesterone acetate (MPA), which potentiated the breast cancer risk [23, 25]. Both estrogenic and combined MHTs preferentially induced ER + breast cancer [28]. These observations support a role for MHTs in the potentiation of preexisting breast cancer cells rather than in the induction of carcinogenesis. In summary, the risk of breast cancer in MHT users was highest for oral estrogen-progestogen formulations used for more than 5 years.

**Table 1:** Menopause hormone therapy modality and associated relative risk of breast cancer [29]

Duration of Treatment	Modality	Relative Risk (95% CI)
1–4 years	Estrogen-only	1.17 (1.10–1.26)
	Estrogen-progestogen	1.60 (1.52–1.69)
5–14 years	Estrogen-only	1.33 (1.28–1.37)
	Estro-progestogen	2.08 (2.02–2.15)
	Topical vaginal administration	1.09 (0.97–1.23)
	Oral administration	1.33 (1.27–1.38)
	Transdermal administration	1.35 (1.25–1.46)
	Sequential modality (Intermittent)	1.93 (1.84–2.01)
	Continuous modality (Daily)	2.30 (2.21–2.40)

Together these data suggest that, if estrogens promote ER + breast cancer growth, exogenous administration of estrogen combined with a progestogen as a MHT in postmenopausal women could increase the risk of breast cancer. In western countries, MHTs are prescribed to almost 12 million women, highlighting the clinical need [28]. The following section aims to provide a critical overview of E4 and to define the potential advantages of E4 over conventional estrogens in the development of a new generation of MHT.

## Estetrol Biology

**Fig. 1:**
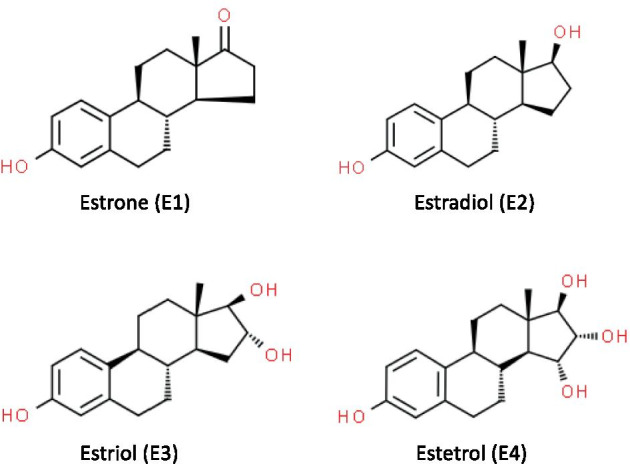
**Biochemical structures of natural estrogens.** Schematic representation of endogenous estrogens, estrone (**E1**), 17β-estradiol (**E2**), estriol (**E3**) and estetrol (**E4**). Structural images were uploaded from ChemSpider (www.chemspider.com) with permission

There are four natural estrogens synthetized in humans, estrone (E1), E2, E3 and E4 (Fig. 1). E4 was discovered in 1965 by the team of Diczfalusy [30]. It is produced exclusively during pregnancy by the liver in both male and female fetuses [30, 31], through 15α- and/or 16α-hydroxylation of E2 or E3 [31]. E4 is detected from the 9^th^ week of gestation in maternal urine and from the 20^th^ week in the maternal plasma. Maternal plasma rates increase during pregnancy and reach 1 ng/ml (3 nM) by the second trimester. E4 fetal plasma levels at term are 12 times higher than those of the mother [32, 33]. Despite work in the 80 s that evaluated maternal E4 level as an index of pregnancy complications and fetus well-being [32, 34, 35], the physiological role of E4 during pregnancy still remains undefined.

In humans, E4 is characterized by a high oral bioavailability of 90% [36], compared to 10% for E2. The human half-life of E4 is 28–32 h [36], compared to 90 min for E2 [14]. In rodents, the bioavailability of E4 is approximately 70% and its half-life is between 2 and 3 h [37]. Both absorption and oral bioavailability are dose-dependent and interindividual plasma variations after oral administration are low [36]. Taken together, the pharmacokinetic parameters of E4 make it suitable for oral use. The hepatic metabolism of E4 is slow in both humans and rodents and is similar across the two species. E4 metabolites are produced through conjugation, mainly methylation, (de)hydroxylation, glucuronidation and sulfation, they are inactive [32, 38] and are rapidly excreted in urine [32, 38–40]. The principal urine metabolite is the Ring D-monoglucuronide, but E4 can also be excreted in an unconjugated form that cannot be reciprocally converted back into E2 or E3 [35, 36].

E4 is selective for the estrogen receptors (estrogen receptor alpha [ERα] and estrogen receptor beta [ERβ]) and binds poorly to other nuclear receptors even at very high concentrations [38]. Although the binding affinity of E4 for both ERα and ERβ is moderate in comparison to E2 [38, 41] (Table 2), its binding affinity for ERα is almost 5 times higher than that of ERβ (Table 2). The interaction of E4 with ERα, at the ligand binding domain is similar to E2 [41]. There is currently no data available on potential binding of E4 to G-coupled protein estrogen receptor (GPER).

**Table 2:** Binding affinity of E2 and E4 for ERα and ERβ—equilibrium dissociation constant (nM) [38]

	ERα	ERβ
E4	4.9 ± 0.6	19 ± 1
E2	0.12 ± 0.03	0.15 ± 0.02

## Estetrol Signaling Pathways

**Fig. 2:**
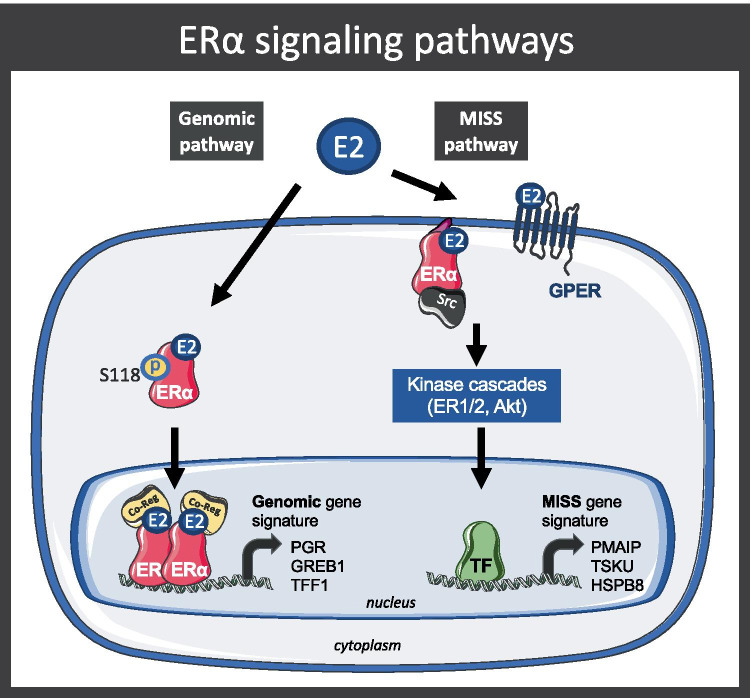
**ERα signaling pathways.** Schematic representation of the genomic pathway and the MISS pathway associated with E2-induced ERα signaling. Abbreviations: S118p, Serine 118 phosphorylated ERα; Co-Reg, co-regulators; TF, transcription factors

In transgenic mouse studies, ERα has been shown to mediate most of the estrogenic actions in organs including the brain, endothelium, mammary gland, vagina, and uterus. ERα has also been shown to control processes such as atheroprotection, vasodilatation, nitric oxide synthesis, endothelial healing, and bone demineralization, and is also involved in the prevention of type-2 diabetes [7, 42–49]. Two distinct signaling pathways are associated with ERα activation (reviewed in [50]): the genomic/nuclear pathway [51] and the non-genomic/extra-nuclear/membrane-initiated steroid signaling (MISS) pathway [50, 52]. The genomic pathway is associated with the transcriptional activity of the nuclear form of the receptor, while the MISS pathway is induced by the membrane-anchored form of ERα or GPER and leads to rapid activation of intracellular signaling cascades (Fig. 2).

**Fig. 3:**
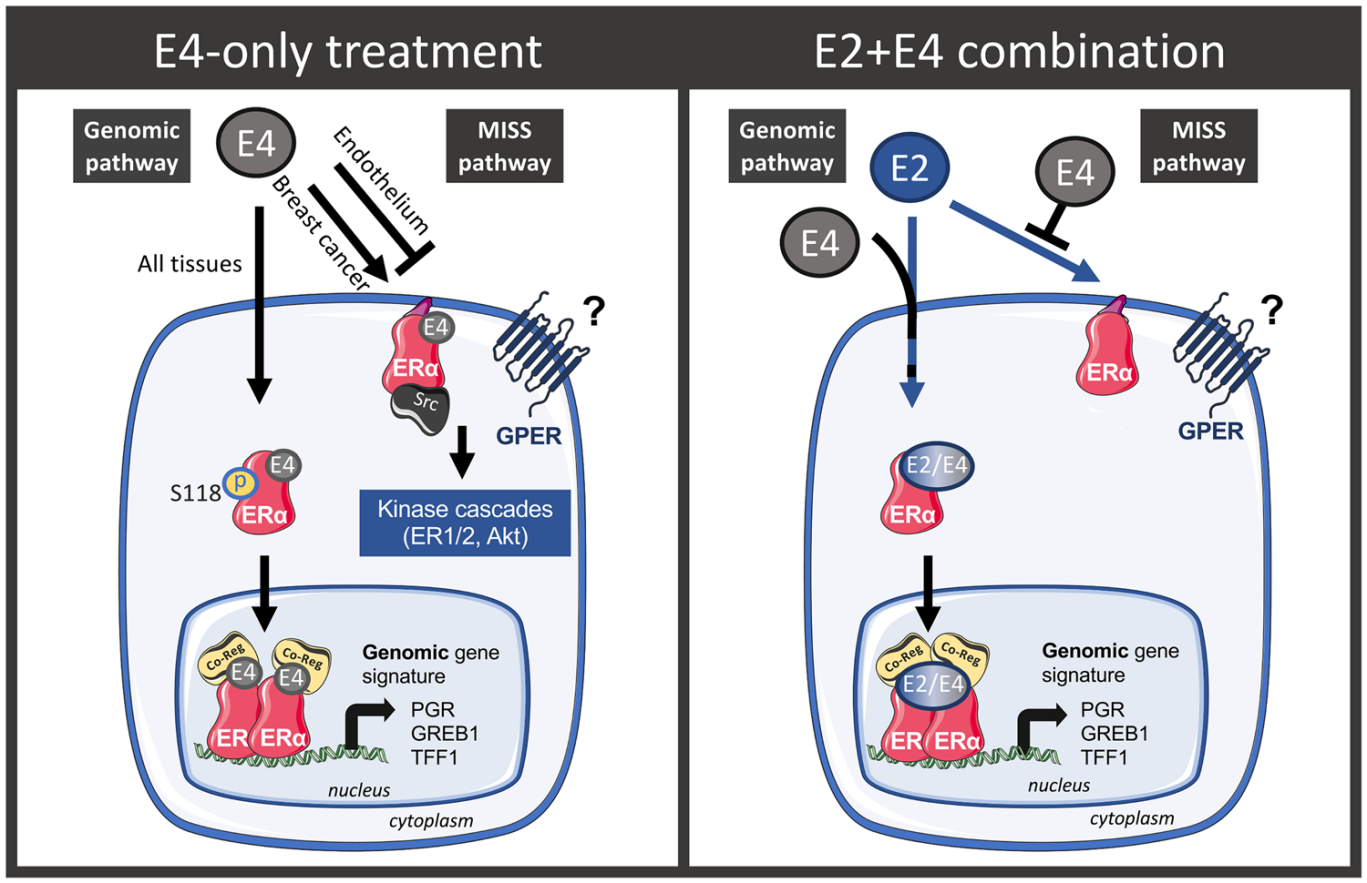
Estrogen signaling pathways induced by E4-only treatment or a combination of E2 + E4

The signaling pathways associated with E4 have been extensively studied in the endothelium in vivo and have been compared to E2-dependent pathways. In rats, E4 has been shown to stimulate vasodilation of arteries via ERα activation [53, 54]. Studies of transgenic mice with mutations in the transactivation function (AF), AF1 or AF2 domain, have revealed that E4 induces atheroprotective effects through the activation of the genomic pathway of ERα [41]. Moreover, E4 exerted a protective effect against neointimal hyperplasia [55], a phenomenon described as being genomic-ERα dependent [56]. E4 was also shown to reduce the effects of angiotensin II and to prevent hypertension in rodents through the genomic-ERα pathway [55]. As predicted by the binding affinity constant (Table 2), the potency of E4 required to activate the genomic-ERα pathway is 50–100 times lower than that of E2. In contrast to E2, E4 did not accelerate endothelial healing in vivo or enhance endothelial nitric oxide synthase (eNOS) activation ex vivo [41, 55]; both of which are actions that have been established to be dependent on the MISS pathway of ERα [44, 57]. In addition, E4 inhibited the action of E2 on endothelial healing and eNOS activation, suggesting E4 antagonizes the E2-induced MISS pathway of ERα [41]. These results support that in the endothelium, E4 is an agonist of the genomic-ERα pathway but an antagonist of the MISS pathway (Fig. 3).

E4 also acts through ERα to promote proliferation and growth of the mouse mammary gland [58], human ER + breast cancer cells [59, 60], patient-derived xenografts (PDX) from ER + breast tumors [60], and endometrial cancer cells [61]. However, in contrast to what was observed in the endothelium, E4 is reported to activate both the genomic-ERα and MISS pathways in breast cancer cells (Fig. 3) [41, 59]. In vitro, E4 stimulated rapid phosphorylation of serine 118 (S118)-ERα, the major phosphorylation site for activation of the receptor by the estrogens [59, 60]. When bound to ERα, E4 promotes the recruitment of the same set of co-regulators as E2, including PELP1 (proline-, glutamic acid-, and leucine-rich protein 1), MED1 (mediator complex subunit 1), SRC1 (steroid receptor coactivator 1), SRC2, SRC3, CBP/p300 (cAMP response element-binding protein), BRD8 (bromodomain-containing protein 8), LCoR (ligand-dependent corepressor), and NRIP1/RIP140 (nuclear receptor-interacting protein 1) [60]. Large scale RNAseq analyses performed on human MCF7 breast cancer cells revealed that, after 24 h incubation, E4 resulted in a similar transcriptomic profile with 97% of genes showing no differential expression in comparison to E2. E4 induced the expression and synthesis of genomic-related targets, such as the progesterone receptor (PR) [59, 60]. In MCF7 cells, E4 activated the MISS signaling pathway, by promoting the interaction between ERα and the tyrosine kinase Src, one of the first step in the MISS pathway [59]. It also induced rapid intracellular signaling cascades, including the MAPK/ERK1/2 and PI3K/AKT pathways. Interestingly, E4 in combination with E2 did not antagonize the E2-induced genomic-ERα pathway when assessed in vitro [41, 59]. Indeed, when E4 was combined with E2, it did not block the binding of ERα to ERE, or the transcription or synthesis of PR. However, E4 resulted in inhibition of the E2-induced MISS pathway in MCF7 cells, as it prevented the interaction between ERα and Src induced by E2 [41]. In breast cancer cells, E4 is an agonist of both the genomic-ERα and the MISS pathway. However, it can also antagonize the E2-induced MISS pathway.

In contrast to endocrine-sensitive ER + breast cancer cells, in endocrine-resistant ER + breast cancer cells E4 induces a distinct ERα-mediated signaling pathway. This pathway results in a gene expression signature associated with the unfolded protein response (UPR) and apoptosis; including downregulation of UPR genes associated with lipid metabolism (MBTPS1), endoplasmic reticulum-associated degradation (HTRA4, SYVN1, HERPUD1), and chaperone proteins involved in pro-survival mechanisms (e.g. SIL1) [61], and upregulation of CEBPB and INHBE, which are associated with high UPR stress. In endocrine-resistant ER + breast cancer cells, this gene signature is associated with a pro-apoptotic profile.

In summary, in all tissues evaluated so far, E4 is an agonist of the genomic-ERα pathway. However, activation of the MISS pathway appears to be tissue dependent. In the endothelium, E4 is an antagonist of the MISS pathway but when used alone in breast cancer cells it is an agonist of the MISS pathway. Interestingly, when combined with E2, E4 prevents activation of the MISS pathway induced by E2 in both the endothelium and in human endocrine-sensitive ER + breast cancer cells (Fig. 3).

## Estetrol and Mammary Gland

To the best of our knowledge, there is only one study that has explored the effect of E4 on the mammary gland in prepubescent mice. It showed that E4 promoted elongation and proliferation of the murine mammary ductal tree in vivo by inducing the growth of prepubertal epithelial ducts and the appearance of terminal end buds (TEBs) [58]*.* Oral administration of E4 at a dose of between 0.3 to 10 mg/kg/day increased the growth of the murine mammary gland, but to a lesser extent than E2. Of note, the levels of E4 detected in the blood were 12–374 times higher than those of E2. Thus, in the murine mammary gland, E4 is considered as a weak estrogen in comparison to E2. This observation was also reported in human breast epithelial (HBE) cells in vitro, where E4 was 100 times less potent at stimulating proliferation of these cells than E2. Interestingly, when HBE cells and mice were treated with a combination of E2 and E4, E4 partially antagonized the strong stimulatory effect of E2 on HBE cell proliferation and murine mammary duct growth [58]. ERα is the receptor that mediates the estrogenic action of E4 on the mammary gland however, the mechanisms of action sustaining the anti-estrogenic effect have not yet been elucidated. Although these observations support the use of E4-based COCs for the prevention of E2-induced mammary gland proliferation in premenopausal women, the combination of E4 and a progestogen has not yet been evaluated in relation to mammary gland biology.

## Estetrol and Breast Cancer

The effect of E4 on tumorigenesis in the breast and on breast cancer progression is complex and remains debated in the literature. Although E4 has primarily been described as pro-apoptotic, it has also been shown to have pro-tumoral effects on breast cancer [59, 62–64]. However, it is important to note the sensitivity status of the breast cancer cells and/or tumors studied with respect to endocrine therapy when interpreting such studies. Visser et al*.* [62]*,* showed an anti-tumoral effect of E4 in a 7,12-dimethylben[a]antracene (DMBA)-induced breast cancer model in rat. In this study, daily oral administration of E4 prevented the initiation of breast tumors in a dose-dependent manner (2.5–10 mg/kg/day) in intact rats with endogenous levels of E2. At high doses (10 mg/kg/day), E4 suppressed breast tumor progression to a similar extent as ovariectomy and more efficiently than tamoxifen treatment [62]. In in vitro experiments performed on a long-term estrogen-deprived (LTED) human MCF7 breast cancer cell line [63] and in endocrine-resistant ER + breast cancer cells [61] E4 had pro-apoptotic properties similar to E2. In endocrine-resistant ER + breast cancer cells, these pro-apoptotic properties were associated with an ERα-dependent UPR [61]. Singer et al*.* [64], reported that when E4 was administrated orally (20 mg/day) for 2 weeks in women with recently diagnosed breast cancer, immunohistochemical assessment of tumors revealed an increased number of apoptotic cells but no modulation of the proliferative marker Ki67, when compared to placebo. This suggests that although E4 induced apoptosis in cancer cells, it does not have an impact on proliferation. In a multicenter, open-label, phase Ib/IIa, dose-escalation (20 mg, 40 mg, and 60 mg E4) study in postmenopausal women with resistant advanced breast cancer who have failed on anti-estrogen treatment with tamoxifen and aromatase inhibitors, E4 had an anti-tumor effect in five of nine patients. However, the three of three patients who received 60 mg E4 exhibited progressive tumor growth [65].

E4 had dose-dependent estrogenic properties, inducing pro-tumoral effects at high doses in human endocrine-sensitive breast cancer cells in vitro and in several breast tumor models in vivo [59, 60, 66]. E4 (10^−8^ M-10^−5^ M) increased the proliferation of MCF7 and T47D human ER + breast cancer cells, but with 50–100 less potency than E2. In addition to these proliferative effects, E4 reduced apoptosis in endocrine-sensitive breast cancer cells and reduced cell cytotoxicity [59]. E4 (10^−9^ M-10^−7^ M) also controlled ER + breast cancer cell (T47D) migration and invasion in a dose-dependent manner, through remodeling of the actin cytoskeleton via phosphorylation of moesin (Thr558); however, the pro-migratory effect of E4 was much lower than that of E2 [67]. In vivo, oral administration of E4 (3–10 mg/kg/day) increased the growth of MCF7 cells engrafted in ovariectomized immunodeficient mice in a dose-dependent manner, although a dose of 0.5 mgE4/kg/day was neutral [59]. These results were corroborated by work in MMTV-PyMT transgenic mice and PDXs from ER + breast cancer [60], which modelled hormonal human treatments by administering steroids to mice in a pattern that closely mimics daily oral steroid exposure in women. In contrast to E2 treatment, experiments using PDXs revealed that long term treatment with E4, delivered continuously over 30 weeks by Alzet pump (0.3 mg/kg/day) to mimic the therapeutic dose used for contraceptive purposes or in the treatment of symptoms due to menopause (15 mg/day), did not increase breast cancer growth. In addition, at this dose E4 treatment did not promote breast cancer metastasis dissemination to the lung in transgenic MMTV-PyMT mice [60]. However, at high pharmacological doses (3 mg/kg/day) exceeding 10 times the therapeutic dose of E4 reported to be required to treat symptoms due to menopause, E4 was pro-tumoral and pro-metastatic. This supratherapeutic dose of E4 (3 mg/kg/day) produces effects similar to the therapeutic dose of E2 required to treat symptoms due to menopause [60]. Interestingly, combined administration of E4 and E2 showed that E4 partially antagonized E2-induced breast cancer cell proliferation and migration in vitro (MCF7, T47D), and MCF7 breast tumor growth in vivo, when used at high doses of 10^–8^-10^−6^ M and 3–10 mg/kg/day, respectively [59, 67, 68]. These observations by Gérard et al*.* [59] in human MCF7 tumor xenografts and those of Visser et al*.* [62] in rat DMBA-induced breast cancer model, highlight the anti-estrogenic action of E4 on the pro-tumoral effect of exogenous and endogenous E2. This anti-estrogenic effect is not related to the antagonistic action of E4 on the genomic-ERα pathway. However, the actual molecular mechanisms employed to sustain this anti-estrogenic effect in endocrine-sensitive breast cancer cells are not yet fully understood.

E4 was also evaluated in combination with progesterone or drospirenone (DRSP) using three complementary endocrine-sensitive breast cancer models [60]: transgenic MMTV-PyMT mice, human MCF7 cell xenografts, and ER + breast cancer PDXs. Progesterone and DRSP were administered to mice in order to match human therapeutic doses. The combination of progesterone or DRSP and E4 (0.3 mg/kg/day) for use as a MHT did not impact tumor growth or metastasis dissemination when compared to E4 used alone. Moreover, the addition of progesterone did not potentiate the pro-tumoral effect observed at a supratherapeutic dose of E4 (3 mg/kg/day). Transcriptomics in MCF7 cells also showed that the addition of progesterone or its analog R5020 to E4 had a limited impact on gene transcription since only few genes were modulated. Nevertheless, several of the genes that were upregulated including, SGK1, FAM105A, FGF18, and TMEM63C, belong to a gene signature associated with progesterone-induced anti-proliferative effects related to good clinical outcomes [69]. These observations show that combining a therapeutic dose of E4 with progesterone or DRSP has a neutral impact on endocrine-sensitive breast cancer models in vivo*.*

Together, these data highlight the complex nature of E4 action in breast cancer. While the estrogenic actions of E4 promote pro-apoptotic features in endocrine-resistant breast cancer, in endocrine-sensitive breast cancers, E4 has a neutral impact even when combined with a progesterone or DRSP at the therapeutic dose for contraception or MHT. However, at doses exceeding this it becomes pro-tumoral in a dose-dependent manner. Notably, when combined with endogenous or exogenous E2, E4 can prevent the pro-tumoral action of E2. The effect of E4 on breast cancer cells is therefore highly dependent on the concentration or dose used and on whether or not it is combined with other estrogens, in particular E2, providing the opportunity to define a safe therapeutic window for use in different clinical applications.

## Estetrol Potential for Therapeutic Indications

Preclinical and clinical studies have identified interesting therapeutic indications for E4, such as contraception, the treatment of symptoms due to menopause, and the treatment of endocrine-resistant breast cancer.

### Contraception

Dose dependent inhibition of ovulation induced by E4 in cycling rats [70–72] was confirmed in a pilot phase II dose-finding study at a dose of 10 mg E4/day; with maximal inhibition seen at 20 mg E4/day [73]. Moreover, E4 treatment was also shown to decrease ovarian (E2) and gonadotropins (LH and FSH) hormone production [73]. The randomized phase II FIESTA study reported a favorable vaginal bleeding pattern and good cycle control associated with 15 mg E4/3 mg DRSP [74]. In addition, E4/DRSP treatment was also shown to be associated with favorable body weight control, well-being, high acceptability, and user satisfaction [75]. Since the combination of E4 and DRSP has been shown to have a neutral impact on breast cancer growth in vivo compared to E2 and progesterone [60], the use of an E4-based COC formulation could be advantageous in women who are concerned about the potential risk of breast cancer associated with conventional COCs. More interestingly, E4 did not increase the synthesis of sex hormone binging globulin (SHBG) [76–78] and had a limited effect on liver factors involved in coagulation, supporting a lower risk of cardiovascular side effects in comparison to E2 [55, 79]. This feature of E4 is possibly the most important advantage of E4-based formulations over conventional COCs.

### Menopause Symptoms

Menopause is associated with the appearance of menopause symptoms, such as vasomotor symptoms (hot flushes and/or night sweats), genitourinary symptoms (vaginal dryness and atrophy, uterine bleeding and sexual dysfunction), urinary symptoms (incontinence and infections), mood change (irritability, anxiety, sadness, hyper-sensibility and depression), and cognitive disturbance [80, 81]. In addition, menopause is also associated with an increased risk of cardiovascular diseases, bone fractures related to osteoporosis, and metabolic complications such as type-2 diabetes [82–85]. Several preclinical and clinical studies have evaluated the impact of E4 on estrogen-sensitive tissues and organs related to menopause symptoms.

In an in vivo model of hot flushes in rats, E4 was shown to suppress vasomotor symptoms in a dose dependent manner [86]. E4 induced the synthesis of allopregnanolone in the brain of castrated rats, which is associated with body temperature regulation [87]. E4 also prevented E2-induced production of this molecule, highlighting the estrogenic and anti-estrogenic properties of E4 [88, 89]. Two clinical studies demonstrated that E4 (at a dose of 2–10 mg/day or 15 mg/day) effectively reduced the frequency and the severity of hot flushes in postmenopausal women [90, 91]. These results support a beneficial effect for E4 in controlling hot flushes.

In rodents, E4 promoted the proliferation of vaginal cells, increased vaginal weight and epithelium height [92], and induced vaginal cornification and maturation [93]. A randomized clinical study evaluating oral administration of E4 (at a dose of 2–40 mg/day) in postmenopausal women also revealed modifications to vaginal cytology, including a decrease in the percentage of parabasal cells and an increase in the quantity of superficial cells [90]. These preclinical and clinical studies show that E4 is associated with protective actions on the vagina and highlight a potential role for E4 in the prevention of vulvo-vaginal atrophy.

Interestingly, E4 attenuated brain injury in a neonatal rat model of hypoxic-ischemic encephalopathy, prevented oxidative stress and enhanced cell proliferation in primary hippocampal neuronal cell cultures in vitro*,* decreased early grey matter loss, and promoted neurogenesis and angiogenesis in vivo [94]. These results show that E4 presents neuroprotective properties and support that E4 could be investigated for the prevention of menopause symptoms related to cognitive function.

In another preclinical study, oral administration of E4 decreased levels of osteocalcin and increased bone density, mineral content, and bone strength in a dose dependent manner in ovariectomized rats [37]. In postmenopausal women, E4 also exerted a dose-dependent decrease in C-telopeptide and osteocalcine, markers of bone resorption and formation, respectively [70, 95]. At higher doses (20 and 40 mg), E4 stimulated bone formation, highlighting the potential use of E4 in the prevention of osteoporosis [70]. These results highlight promising clinical benefits and a potential role for E4 on bone fractures and osteoporosis risk.

Additional positive effects of E4 were also reported on menopause-associated risks, including metabolic disorders and cardiovascular issues. In mouse models, E4 reduced body weight gain, improved glucose tolerance, prevented obesity [96], and associated disorders such as atherosclerosis and steatosis [53]. Preclinical and clinical studies revealed that E4 has several potential vascular advantages (reviewed in [55]), including the prevention of angiotensin-II-dependent hypertension and neointimal hyperplasia, while having minimal impact on hemostasis, fibrinolysis, angiotensinogen, triglycerides, and cholesterol. Although E4 did not enhance eNOS action in murine adult aorta [41, 55], it favored flow-induced vasodilation [53, 54], an important vasculoprotective action of estrogen. In addition, E4 also prevented atherosclerosis in a dose-dependent manner in mice [41].

Importantly, preclinical and clinical studies demonstrated that E4 induces uterotrophic activity and acts as an agonist on the endometrium [41, 90]. It has also been shown to increase mouse uterine wet weight [37, 93, 97], epithelial proliferation, and height of the epithelium and stromal compartments [41] at a MHT therapeutic dose [60]. In rats, E4 increased the volume of luminal fluids, protein content, and the activity of alkaline phosphatase [97]; all markers of the estrogenic uterine response. Moreover, it induced major histological modifications, including the synthesis of the PR [98]. In postmenopausal women, E4 (10 mg/day) increased the thickness of the endometrium, highlighting its estrogenic action [90]. The addition of DRSP to E4 (5 or 10 mg/day) decreased E4-induced endometrial thickening in women [73]. These results support the addition of a progestogen to E4 formulations for MHT to protect the endometrium of non-hysterectomized women from hyperplasia and cancer.

Assessing the risk of breast cancer associated with E4 use in postmenopausal women is a long-term effort and can only be conducted with decades of patient follow-up. Nevertheless, as detailed in the previous section of this review, preclinical data obtained from several endocrine-sensitive breast cancer models, including PDX, revealed that E4 has a neutral impact on breast cancer growth and metastasis dissemination to the lung when used in mice at a dose (0.3 mg/kg/day) that corresponds to the steady-state obtained by once-a-day (15 mg E4/day) oral treatment in women. This neutral effect is not impacted by the addition of progesterone or DRSP [60]. These observations give E4-based formulations an advantage over E2-based MHTs and have important clinical implications as they highlight the possibility of developing a combined estrogen, progestogen MHT that could have a positive impact on breast cancer risk.

### Breast Cancer Treatment

Acquired endocrine resistance is a major cause of relapse in ER + breast cancer and therapeutic strategies to help overcome this are of the utmost importance. Several studies have suggested the potential of estrogen-based therapies in the treatment of advanced endocrine-resistant breast cancer. However, there is a reluctance to use E2 due to the potential for adverse effects, especially thromboembolism [99–104]. The pro-apoptotic properties of E4 on endocrine-resistant breast cancer demonstrated in vitro [61] and in clinical trials [64, 65], in addition to the limited effect of E4 on liver factors involved in coagulation [79], support a potential role for E4 in the treatment of advanced endocrine-resistant breast cancer in postmenopausal women.

## Conclusions and Perspectives

Of the natural estrogens, E4 is a unique native fetal estrogen with selective tissue actions that offers novel therapeutic opportunities for indications including, contraception, the treatment of symptoms due to menopause, and the treatment of endocrine-resistant advanced breast cancer.

The safety data for E4 in the breast are promising, nevertheless further progress in this field is expected, especially regarding the mechanisms of action of this natural estrogen on the mammary gland and breast cancer. A recent publication emphasized that, in addition to the genomic-ERα pathway [105], the MISS-ERα pathway also plays a role in promoting intercellular communication during mammary gland development [106]. Since E4 is characterized as an antagonist and an agonist of the MISS pathway depending on the tissue, it is important to fully characterize the molecular impact of E4 on mammary gland biology. In breast cancer, preclinical and clinical studies have shown that E4 formulations have both pro-tumoral and pro-apoptotic effects depending on the dose and whether or not it is combined with endogenous or exogenous E2. The molecular mechanisms leading to the anti-estrogenic action of E4 in particular, remain to be fully elucidated.

In conclusion, E4 does not meet every characteristic of the ideal estrogen. However, in comparison to other conventional estrogens, E4 does meet several important criteria. E4 could be considered as a friend of the mammary gland, even when combined to progesterone or DRSP to protect the endometrium of non-hysterectomized women, since it remains neutral on preclinical models of breast cancer at a dose that is effective at preventing hot flushes; a symptom of menopause that arises in 80% of postmenopausal women. However, at high dose, E4 remains a foe of the mammary gland highlighting the importance of exerting extreme caution when determining the dose required for management versus prevention of mammary side effects. E4 also displays cardioprotective features against atherosclerosis and has a limited impact on liver factors involved in coagulation, supporting a lower risk of thromboembolic events and thromboembolism. These features support a safer profile in terms of breast cancer risk and thromboembolism risk making E4 a safer estrogenic treatment option for women.
